# Chronic Kidney Disease with Mild and Mild to Moderate Reduction in Renal Function and Long-Term Recurrences of Atrial Fibrillation after Pulmonary Vein Cryoballoon Ablation

**DOI:** 10.3390/jcdd9050126

**Published:** 2022-04-21

**Authors:** Giuseppe Boriani, Saverio Iacopino, Giuseppe Arena, Paolo Pieragnoli, Roberto Verlato, Massimiliano Manfrin, Giulio Molon, Giovanni Rovaris, Antonio Curnis, Giovanni Battista Perego, Antonio Dello Russo, Maurizio Landolina, Marco Vitolo, Claudio Tondo, on behalf of the 1STOP ClinicalService Investigators

**Affiliations:** 1 Cardiology Division, Department of Biomedical, Metabolic and Neural Sciences, University of Modena and Reggio Emilia, Policlinico di Modena, 41121 Modena, Italy; marco.vitolo@unimore.it; 2 Electrophysiology Unit, Maria Cecilia Hospital, GVM Care & Research, 48033 Cotignola, Italy; iacopino@iol.it; 3 Ospedale delle Apuane, 54100 Massa, Italy; giuseppe.arena@uslnordovest.toscana.it; 4 Ospedale Careggi, University of Florence, 50121 Firenze, Italy; pieragnolip@aou-careggi.toscana.it; 5 ULSS 6 Euganea, Ospedale di Camposampiero-Cittadella, 35013 Cittadella, Italy; roberto.verlato@gmail.com; 6 Ospedale San Maurizio, 39100 Bolzano, Italy; massimiliano.manfrin@sabes.it; 7 IRCCS Sacro Cuore don Calabria, 37024 Negrar, Italy; giulio.molon@sacrocuore.it; 8 ASST San Gerardo di Monza, 20900 Monza, Italy; gi.rovaris@gmail.com; 9 ASST degli Spedali Civili, 25123 Brescia, Italy; antonio.curnis@libero.it; 10 Istituto Auxologico Italiano IRCCS, Ospedale San Luca, 55100 Milano, Italy; perego@auxologico.it; 11 Ospedali Riuniti Torrette di Ancona, 60126 Torrette, Italy; antonio.dellorusso@gmail.com; 12 ASST Ospedale Maggiore, 26013 Crema, Italy; maurizio.landolina02@gmail.com; 13 Clinical and Experimental Medicine PhD Program, University of Modena and Reggio Emilia, 41121 Modena, Italy; 14 Department of Clinical Electrophysiology & Cardiac Pacing, Heart Rhythm Center, Monzino Cardiac Center IRCCS, 20122 Milan, Italy; claudio.tondo@cardiologicomonzino.it; 15 Department of Biochemical, Surgical and Dentist Sciences, University of Milan, 20122 Milan, Italy

**Keywords:** atrial fibrillation, cryoablation, chronic kidney disease, catheter ablation, rhythm control

## Abstract

The aim of this research was to evaluate if patients with chronic kidney disease (CKD) and mild or mild to moderate depression of renal function have an increased risk of atrial fibrillation (AF) recurrences after cryoballoon (CB) ablation. We performed a retrospective analysis of AF patients undergoing pulmonary vein isolation (PVI) by CB. The cohort was divided according to the KDIGO CKD-EPI classification into a (1) normal, (2) mildly decreased, or (3) mild to moderate reduction in estimated glomerular filtration rate (eGFR). Freedom from AF recurrences was the primary endpoint. A total of 1971 patients were included (60 ± 10 years, 29.0% females, 73.6% paroxysmal AF) in the study. Acute success and complication rates were 99.2% and 3.7%, respectively, with no significant differences among the three groups. After a follow-up of 24 months, AF recurrences were higher in the mildly and mild to moderate CKD groups compared to the normal kidney function group (23.4% vs. 28.3% vs. 33.5%, *p* < 0.05). Mild to moderate CKD was an independent predictor of AF recurrences after the blanking period (hazard ratio:1.38, 95% CI 1.02–1.86, *p* = 0.037). In conclusion, a multicenter analysis of AF patients treated with cryoablation revealed mild to moderate reductions in renal functions were associated with a higher risk of AF recurrences. Conversely, the procedural success and complication rates were similar in patients with normal, mildly reduced, or mild to moderate reduction in eGFR.

## 1. Introduction

Atrial fibrillation (AF) is frequently associated with comorbidities that influence the course of AF, along with time, patient symptoms, thromboembolic risk, and patient outcome [1,2,3]. Among the comorbidities more frequently associated with AF, chronic kidney disease (CKD) has special value, in consideration of the existing pathophysiological links, the influence of patient management, and treatments [4]. However, evidence gaps on the best strategies to adopt at specific stages of CKD are still present [1,4,5,6]. As a matter of fact, the stages of CKD, as proposed by Kidney Disease: Improving Global Outcomes (KDIGO) [4,7,8] are an important reference for clinical decisions and patients outcome in many cardiac diseases, both for medical and interventional treatments [9,10,11]. Catheter ablation with pulmonary veins isolation (PVI) is currently a valuable treatment option for applying a rhythm-control strategy to AF patients, and also as first-line treatment [2,12,13]. Two approaches have been most widely used in daily practice. These are radiofrequency ablation and cryoablation, and these two approaches present some differences in short- and long-term outcomes [12,13,14]. In the case of advanced CKD or dialysis, the risk of AF recurrences after AF ablation is significantly higher than in patients with less advanced CKD [15]. However, the impact on AF recurrences of less advanced stages of CKD remains unclear, even though the less advanced stages are more frequently represented in the population of AF patients considered as candidates for AF ablation. 

The aim of the present research is to evaluate the follow up of AF patients treated with cryoballoon (CB) ablation for PVI, in order to assess if patients with mild or mild to moderate depression of renal function are associated with an increased risk of AF recurrences at mid to long-term, as compared with patients with normal renal function.

## 2. Materials and Methods

### 2.1. Research Design and Patient Population

Patients with AF who had undergone CB catheter ablation for PVI, and who were participating in the One Shot TO Pulmonary vein isolation (1STOP) project within the One Hospital ClinicalService (OHCS) were considered for the present analysis. The project aims to improve the quality of diagnostic and therapeutic strategies through the use of CB ablations in clinical practice. It consists of a shared environment for the prospective collection, management, analysis, and reporting of data from patients in whom Medtronic devices have been implanted. Patients are prospectively followed by 25 Italian cardiology centers according to clinical practice and guidelines through standard in-hospital visits. An independent scientific committee of physicians prospectively identifies key clinical questions on a yearly basis for analysis and publication. A charter assigns the ownership of data to the centers and governs the conduct and relationship of the scientific committee and Medtronic. The project was approved by each site’s Medical Ethics Committee or medical director, and it conforms to the principles outlined in the Declaration of Helsinki. Each patient provided informed consent for data collection and analysis. 

The present study is a retrospective analysis of a series of 1971 prospectively enrolled patients. Patients were included if they were treated for AF through PVI with one of four generations of the CB catheter, had a clinical follow-up reported in the OHCS database, had not had any previous PVI or previous AF ablations, and had creatinine data recorded before the ablation. The total patient cohort was divided into the following four groups according to the KDIGO CKD-EPI classification: (1) normal kidney function, meaning a normal or increased eGFR (≥90 mL/min/1.73 m^2^); (2) mildly decreased kidney function, meaning a mild reduction in eGFR (60–89 mL/min/1.73 m^2^); (3) mild to moderate CKD, meaning a mild to moderate reduction in eGFR (30–59 mL/min/1.73 m^2^); (4) severe CKD, meaning a severe reduction in eGFR (15–29 mL/min/1.73 m^2^). Patients with severe CKD (eGFR 15–29 mL/min/1.73 m^2^) were excluded from the outcome analysis because they had been clinically judged as a small, non-representative sample of the CB ablation population.

A detailed description of the ablation procedure protocol and periprocedural management has been previously described [16]. In brief, each center used its standard of care practices and approaches during the cryo-ablation procedure. Patients were generally sedated using either general anesthesia or conscious sedation. Cryoablations were performed using a 23 and/or 28 mm cryoballoon. The number of freeze applications and length of individual freezes were determined by the operators according to the centers’ standard of care usage. Acute PVI success was defined as electrical conduction isolation confirmed by bidirectional block. 

### 2.2. Follow-Up and Data Collection

The baseline assessment at procedure included the collection of demographic information, medical history, and data on procedural characteristics and procedure duration. Follow-up visits were made in accordance with the clinical practice of each center and included the assessment of the patient’s AF-related symptoms, ECG or Holter monitoring examination, and drug therapy assessment.

### 2.3. Research Objectives

The main objective of our research is to evaluate the clinical efficacy (i.e., freedom from AF recurrences) of a single PV procedure in patients with and without a history of mild to moderate CKD. Ablation is defined as successful in the absence of asymptomatic or symptomatic atrial arrhythmias lasting more than 30 s, as identified by Holter monitoring or AF detected at a 12-lead ECG after the blanking period (3 months since the date of ablation procedure). The occurrence of AF during the first 3 months after the AF ablation was defined as an early recurrence of AF (ERAF) and, as such, was excluded from the main analysis. CKD has been calculated according to the patient’s eGFR class, using the KDIGO CKD-EPI method, and considering the patient’s age and the creatinine value collected at the time of the ablation. The acute success rate of PV has been defined as the ratio between the number of effectively isolated PVs and the number of target PVs.

### 2.4. Statistical Analyses

Baseline characteristics and clinical and procedural data have been summarized for the three considered patient groups according to the KDIGO CKD-EPI classification. Continuous variables were reported as mean and standard deviation (SD) or as median and interquartile range (IQR). Categorical variables were reported as counts and percentages. Patients with normal kidney function were compared with those presenting mildly decreased kidney function and those with mild to moderate CKD using Wilcoxon’s test for continuous variables, and the chi-square test or Fisher’s exact test for extreme proportions in case of categorical variables, as appropriate. Annual rates of AF recurrence were compared by means of a mixed Poisson model. The analyses of time-to-first event were described using Kaplan–Meier curves and compared between the groups with the log-rank test. The follow-up duration (months) was computed from the date of the CB catheter ablation to the date of the last available follow-up or date of the event. To find predictors of AF recurrences, a Cox regression was used for both univariable and multivariable analyses, and the proportional hazard hypothesis was tested. The hazard ratios (HRs) and 95% confidence intervals (CIs) were estimated for all potential predictors. The multivariable Cox regression model used stepwise selection with entry = 0.30 and stay = 0.05 criteria, respectively, and AF as the dependent variable. Statistical tests were based on a two-sided significance level of 0.05. The Bonferroni method has been used to adjust for multiple comparisons. According to Bonferroni correction, post-hoc comparisons between patient groups were considered statistically significant for *p*-values < 0.025. The SAS software, version 9.4, (SAS Institute Inc., Cary, NC, USA) was used to perform statistical analyses.

## 3. Results

### 3.1. Patient Population

Out of the 1976 patients who underwent CB catheter ablation for AF treatment from May 2010 to May 2021, as extracted from the One Hospital ClinicalService 1STOP project, 1971 (99.7%) had non-severe CKD (i.e., eGFR ≥ 30 mL/min/1.73 m^2^), and, as such, were included in the analysis. The patients were then classified according to the following KDIGO CKD-EPI definition: 774 (39.3%) as normal kidney function patients, 1015 (51.5%) as mild decreased kidney function patients, and 182 (9.2%) as mild to moderate CKD patients. The baseline clinical characteristics are shown in Table 1 for the whole population, as well as by kidney disease status. In brief, the mean age was 60 ± 10 years, 29.0% of patients were female, 73.6% had a history of paroxysmal AF only, while 52.5% patients suffered from hypertension. Patients with mild to moderate CKD were older (69 years vs. 56 years and 64 years in the normal and in the mildly decreased kidney function groups, respectively, *p* < 0.001), more likely to be female (45.1% vs. 23.9% vs. 30.0%, respectively, *p* < 0.001), more frequently had hypertension (68.1% vs. 42.0% vs. 57.6%, respectively, *p* < 0.001) and had a higher risk of CHA_2_DS_2_-VASC (93.9% vs. 77.6% vs. 54.6%) or any underlying heart disease (32.4% vs. 17.6% vs. 23.7%, *p* ≤ 0.001). 

### 3.2. Procedural Data

Index procedure data and acute results are summarized in Table 2. The majority of the patients were treated with a second-generation cryoballoon (87.2%, Arctic Front Advance, Medtronic, Dublin, Ireland). First, third and fourth-generation cryoballoons were used, respectively, in 1.9%, 4.1%, and 6.7% of the total cohort. Overall, the average acute success rate was 99.2% for the total population, with no significant differences among the three analysis groups (*p* = 0.519). Despite the fac that no statistically significant differences were found when the total procedure duration has been compared by kidney status (*p* = 0.142), we have observed slightly increased ablation times for the mild to moderate CKD group when compared with the normal kidney function patients (median time: 20 min for mildly decreased kidney function, 18 min for mild to moderate CKD vs. 16 min for normal kidney function patients, *p* = 0.004). No differences were found in the risk of acute complications (3.5% vs. 4.0% vs. 2.7%, respectively, in the normal kidney function patients, the mildly decreased kidney group, and the mild to moderate CKD group, *p* = 0.641). 

### 3.3. AF Recurrences after the Blanking Period

**Figure 1:**
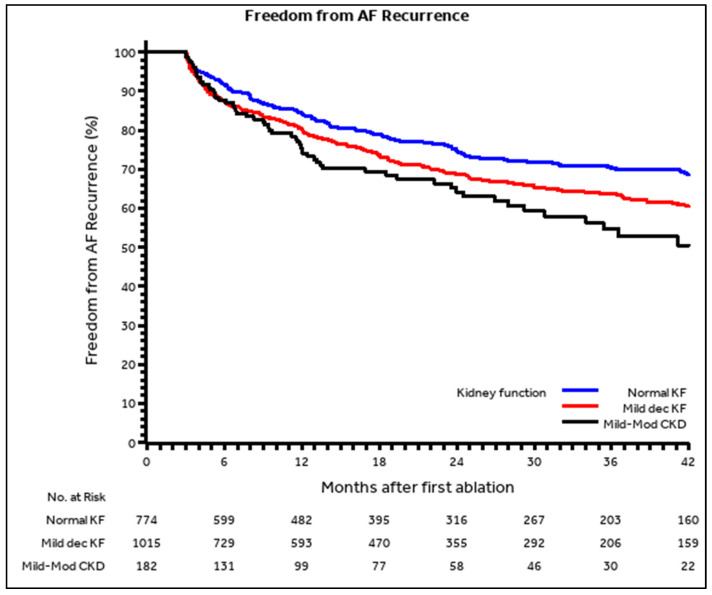
Kaplan–Meier curves. Freedom from AF recurrence according to kidney function. Legend: “Normal KF” = “Normal Kidney Function”; “Mild dec KF” = “Mildly decreased Kidney Function”; “Mild-Mod CKD” = “Mild to Moderate CKD”.

Overall, the median follow-up since the ablation procedure was 24 (Q1–Q3: 10–42) months with no differences among the three analyzed groups [25 (Q1–Q3: 11–46) months vs. 23 (Q1–Q3: 10–39) months vs. 22 (Q1–Q3: 8–39) months, respectively, in the normal kidney function, mildly decreased kidney function, and mild to moderate CKD group, *p* = 0.092]. During the follow-up, AF recurrence was reported in 529 patients, 181 (23.4%, 11.3 patients/year) in the normal kidney function group, 287 (28.3%, 14.3 patients/year) in the mildly decreased kidney function group, and 61 (33.5%,18.3 patients/year) in the mild to moderate CKD group (post-hoc *p* = 0.003 for mildly decreased vs. normal kidney function, and post-hoc *p* ≤ 0.001 for mild to moderate CKD vs. normal kidney function). The Kaplan–Meier 3 year freedom from AF recurrence were 70.2% (66.0–73.9%) vs. 63.6% (59.8–67.2%) vs. 54.7% (44.5–63.7%), respectively, (overall *p* = 0.001; post-hoc *p* < 0.001 for mild to moderate CKD vs. normal kidney function, and post-hoc *p* = 0.007 for mildly decreased vs. normal kidney function, Figure 1).

### 3.4. Predictors of AF Recurrences after the Blanking Period

Predictors of freedom from AF after the CB catheter ablation are presented in Table 3, at the univariate and multivariable analyses. Additionally, the observed freedom from repeat ablations following index procedure at the 42 month follow-up was 90.1% (86.9–92.5%) for the normal kidney function recipient, 84.6% (81.0–87.6%) for those with mildly decreased kidney function, and 84.2% (74.5–90.4%) for mild to moderate CKD patients, respectively (*p* = 0.096). 

### 3.5. Early Recurrence of AF (During the Blanking Period)

Overall, ERAF in the first three months after ablations recurred in 161 (8.2%) patients. Notably, data from the group with the worst renal function highlighted the highest rate of ERAF (5.9% vs. 9.3% vs. 11.5%, post-hoc *p* = 0.010 for mildly decreased vs. normal kidney function, and post-hoc *p* = 0.008 for mild to moderate CKD vs. normal kidney function, respectively). However, at multivariable analysis (Table 4), renal function was not found to be a variable independently associated with early recurrences, differently from a non-paroxysmal AF pattern and a time period from the first episode of atrial arrhythmia longer than 12 months.

## 4. Discussion

Our analysis shows that in AF patients treated with the technique of cryoballoon ablation, even mild to moderate reductions in renal functions are associated with a higher risk of AF recurrences after the blanking period. Conversely, the procedural success rates and acute complications were similar in subgroups with a normal, mildly reduced, or mild to moderate reduction in eGFR. 

In our multicenter study that included around 2000 patients, a worse renal function was associated with a different clinical profile, as compared with patients with normal renal function, with patients who had a lower eGFR being older, more frequently affected by underlying heart disease, and with a higher CHA_2_DS_2_VASc score. However, at multivariable analysis, mild to moderate CKD (eGFR 30–59 mL/min/1.73 m^2^) was significantly and independently associated with AF recurrences after the blanking period. Other factors associated with the risk of recurrences were a period from the first episode of AF longer than one year, and AF ablation performed for persistent, i.e., non-paroxysmal, AF.

Our findings are noteworthy because they are derived from a multi-center collection of data from Italian centers using cryoballoon ablation, a technique widely implemented in daily practice [13,17,18,19]. A report from China dealing with patients treated with either radiofrequency ablation or cryoballoon ablation in a single center (Guangzhou, China) highlighted a stepwise increase in the risk of AF recurrence as CKD worsened up to severe CKD, using the Modification of Diet in Renal Disease (MDRD) equation for estimating the eGFR [20]. It is well known that the epidemiology, presentation, and management of AF differs around the world, with specific differences between Asia and Europe [21,22,23,24,25]. Therefore data from different geographies are useful in order to provide a more detailed picture of the ways to approach and control AF, in the perspective of health technology assessments [26]. 

Balloon cryoablation is now a well-established technique, with some advantage in terms of procedure time and need for re-ablation as compared to radiofrequency ablation [14,27,28]. Our results suggest that, from a clinical point of view, even a mild to moderate reduction in eGFR has an impact on outcome, in line with the evidence that patient profile is a major determinant of long-term outcome in AF patients and, thus, requires individualized decision-making [1,3,29,30,31,32,33]. 

Advanced CKD and AF share common risk factors with many pathophysiological inter-relationships, with the most powerful influence on AF and clinical outcomes being renal dysfunction and progression in end stage renal disease including dialysis [4,34,35]. 

From this perspective, our research is quite reassuring, since it is focused on patients’ mild to moderate reduction of eGFR using cryoballoon technology. The assessment of renal function should be done in accordance with the consensus documents and with formulas for estimating renal function that are more appropriate and validated [7]. Despite the fact that the dosing of direct oral anticoagulants has been validated on the basis of the Cockroft–Gault formula for estimating creatinine clearance [36], CKD-EPI, the formula used in the present analysis, is widely accepted for appropriate categorization of renal function, and has the advantage of being easily accessible, in contrast to the cystatin-based equations which have been shown to be more accurate [37,38,39]. Patients with advanced CKD have a more complex clinical profile and, therefore, a comprehensive approach is required, in line with the ABC pathway suggested by the most recent guidelines, taking care of the entire patient profile and comorbidities [32,40,41,42,43,44].

Our results show that the efficacy of cryoablation in preventing AF recurrences is higher when the procedure is performed within one year of the first AF episode. This is in line with current recommendation of not considering AF ablation as a last-resort therapeutic strategy, but rather to propose it before the development of atrial dilatation and atrial cardiomyopathy [45,46,47,48], which imply a reduced effectiveness of the intervention. Recent data on AF ablation [12,49,50] and more general data from EAST AF trial indicate that the success of rhythm control strategies is markedly influenced by the time from AF clinical detection [51,52,53]. It will be matter of additional investigation to assess the influence of asymptomatic AF episodes, which could be present in a substantial number of patients, with no differences in the impact on hard clinical outcomes, such as stroke [54,55,56,57]. Interestingly, our findings show that less than 10% of patients presented ERAF, confirming that ERAF are rare and are a strong predictor of AF recurrence in the follow-up, above all when it occurs >30 days after the ablation [58].

The present research has specific limitations that should be acknowledged. This a retrospective analysis of prospectively collected data and, as such, has some limitations linked to its observational nature. A specific limitation is the absence of data regarding the contrast medium used in each procedure. Data presented do not imply causality, but rather describe an association, since we cannot exclude the influence of non-measured confounding forces. However, research endpoints were pre-specified.

## 5. Conclusions

In a multicenter analysis on AF patients treated with the technique of cryoballoon ablation, even mild to moderate reductions in renal functions are associated with a higher risk of AF recurrences after the blanking period. Conversely, the procedural success rates and acute complications were similar in subgroups with a normal, mildly reduced, or mild to moderate reduction in eGFR.

## Figures and Tables

**Table 1:** Baseline characteristics.

	Total *n* = 1971	Normal Kidney Function (eGFR: ≥90 mL/min/1.73 m^2^) *n* = 774	Mildly Decreased Kidney Function (eGFR: 60–89 mL/min/1.73 m^2^) *n* = 1015	Mild to Moderate CKD (eGFR: 30–59 mL/min/1.73 m^2^) *n* = 182	*p*-Value
Age at first ablation (years), mean ± SD	60.0 ± 10.4	54.6 ± 10.5	62.7 ± 8.7	68.2 ± 6.7	<0.001 ^1,2^
Female sex, *n* (%)	572 (29.0%)	185 (23.9%)	305 (30.0%)	82 (45.1%)	<0.001 ^1,2^
BMI, mean ± SD	27.1 ± 4.2	27.1 ± 4.4	27.2 ± 3.9	27.4 ± 4.8	0.768
Paroxysmal, *n* (%)	1450 (73.6%)	608 (78.6%)	710 (70.0%)	132 (72.5%)	0.001
Months from first AF diagnosis, median (Q1–Q3)	26 (12–60)	24 (12–60)	26 (12–60)	36 (12–96)	0.386
Previous therapy using >2 AADs, *n* (%)	723 (41.5%)	266 (38.8%)	372 (41.8%)	85 (51.2%)	0.014 ^1,2^
Diabetes, *n* (%)	114 (6.2%)	40 (5.5%)	64 (6.7%)	10 (5.9%)	0.551
Hypertension, *n* (%)	1030 (52.5%)	324 (42.0%)	582 (57.6%)	124 (68.1%)	<0.001 ^1,2^
History of stroke or TIA, *n* (%)	85 (4.4%)	27 (3.5%)	45 (4.5%)	13 (7.2%)	0.090
No underlying heart disease, *n* (%)	1525 (77.9%)	634 (82.4%)	768 (76.3%)	123 (67.6%)	<0.001 ^1,2^
CHA_2_DS_2_VASc ≥1 (male) or ≥2 (female), *n* (%)	1261 (69.8%)	393(54.6%)	713 (77.3%)	155 (93.9%)	<0.001 ^1,2^
Left atrial diameter (mm), mean ± SD	41.8 ± 6.1	40.9 ± 6.2	42.3 ± 6.0	42.8 ± 6.1	<0.001 ^1^
Left ventricular ejection fraction (%),mean ± SD	58.7 ± 6.8	59.4 ± 6.2	58.5 ± 6.9	56.9 ± 8.5	<0.001 ^1^

Legend: AAD = antiarrhythmics drugs; AF = atrial fibrillation; BMI = body mass index; CKD = chronic kidney disease; eGFR = estimated glomerular filtration rate; SD = standard deviation; TIA = transient ischemic attack. ^1,2^ Post-hoc comparisons are as follows: (1) normal kidney function vs. mild to moderate CKD; (2) mildly decreased kidney function vs. mild to moderate CKD.

**Table 2:** Procedural characteristics and acute procedural complications.

	Total *n* = 1971	Normal Kidney Function (eGFR: ≥90 mL/min/1.73 m^2^) *n* = 774	Mildly Decreased Kidney Function (eGFR: 60–89 mL/min/1.73 m^2^) *n* = 1015	Mild to Moderate CKD (eGFR: 30–59 mL/min/1.73 m^2^) *n* = 182	*p*-Value
**Procedural characteristics**					
Procedure duration (min), mean ± SD	107.3 ± 46.8	105.2 ± 45.2	108.0 ± 47.5	112.3 ± 49.5	0.142
Fluoroscopy duration (min), mean ± SD	28.8 ± 16.4	29.4 ± 17.5	28.4 ± 15.5	28.8 ± 17.0	0.666
Ablation time (min), mean ± SD	28.4 ± 55.0	26.2 ± 58.6	28.4 ± 38.8	38.8 ± 99.8	0.0041
Effective PVI, *n* (%)	1956 (99.2%)	769 (99.4%)	1007 (99.2%)	180 (98.9%)	0.519
Pre-ablation sinus rhythm, *n* (%)	1415 (74.1%)	587 (78.2%)	709 (72.2%)	119 (67.6%)	0.0021
Cardioversion, *n* (%)	493 (25.0%)	172 (22.2%)	266 (26.2%)	55 (30.2%)	0.0371
Post-ablation sinus rhythm, *n* (%)	1841 (97.2%)	726 (97.4%)	950 (97.2%)	165 (95.9%)	0.517
**Acute procedural complications**					
Patients with at least one complication, *n* (%)	73 (3.7%)	27 (3.5%)	41 (4.0%)	5 (2.7%)	0.641
Transient Diaphragmatic Paralysis, *n* (%)	41 (2.1%)	15 (1.9%)	24 (2.4%)	2 (1.1%)	0.594
Permanent Diaphragmatic Paralysis, *n* (%)	2 (0.1%)	1 (0.1%)	1 (0.1%)	0 (0.0%)	1.000
Pericardial effusion, *n* (%)	7 (0.4%)	3 (0.4%)	3 (0.3%)	1 (0.5%)	0.620
Cardiac Tamponade, *n* (%)	4 (0.2%)	1 (0.1%)	2 (0.2%)	1 (0.5%)	0.415
AV Fistula, *n* (%)	4 (0.2%)	2 (0.3%)	1 (0.1%)	1 (0.5%)	0.300
Femoral pseudo-aneurism, *n* (%)	2 (0.1%)	1 (0.1%)	1 (0.1%)	0 (0.0%)	1.000
Stroke, *n* (%)	0 (0.0%)	0 (0.0%)	0 (0.0%)	0 (0.0%)	----
TIA, *n* (%)	2 (0.1%)	1 (0.1%)	1 (0.1%)	0 (0.0%)	1.000
Hematoma, *n* (%)	5 (0.3%)	2 (0.3%))	3 (0.3%)	0 (0.0%)	1.000
Other minor complications, *n* (%)	7 (0.4%)	2 (0.3%)	5 (0.5%)	0 (0.0%)	0.851

Legend: SD = standard deviation; PVI = pulmonary vein isolation; TIA = transient ischemic attack; AV fistula = Atrioventricular fistula. Post-hoc comparisons are as follows: (1) normal kidney function vs. mild to moderate CKD; (2) mildly decreased kidney function vs. mild to moderate CKD.

**Table 3:** Predictors of AF recurrences after the blanking period.

**Univariate Analysis**		
	**HR (95% CI)**	***p*-Value**
Mild to moderate CKD (eGFR 30–59 mL/min/1.73 m^2^) vs. eGFR 60 mL/min/1.73 m^2^ or higher	1.40 (1.06–1.83)	0.017
Female gender	1.04 (0.86–1.26)	0.674
Age at first ablation (years) ≥ 65	1.12 (0.94–1.34)	0.201
Paroxysmal AF	0.76 (0.63–0.92)	0.004
Months from the first episode of atrial arrhythmia > 12 months	1.27 (1.03–1.58)	0.025
Number of tested AAD ≥ 2	1.26 (1.05–1.51)	0.014
Underlying heart disease	1.08 (0.88–1.33)	0.466
Hypertension	1.09 (0.91–1.30)	0.349
CHA_2_DS_2_VASc ≥ 1 (male) or ≥2 (female)	1.09 (0.90–1.33)	0.371
LVEF (%, continuous)	0.99 (0.98–1.01)	0.265
**Multivariable Analysis**		
Mild to moderate CKD (eGFR 30–59) vs. eGFR 60 mL/min/1.73 m^2^ or higher	1.38 (1.02–1.86)	0.037
Female gender	0.99 (0.81–1.22)	0.957
Age at first ablation (years) ≥ 65	1.05 (0.86–1.27)	0.626
Paroxysmal AF	0.78 (0.64–0.96)	0.019
Months from the first episode of atrial arrhythmia > 12 months	1.27 (1.03–1.57)	0.026

Legend: AAD = antiarrhythmics drug; AF = atrial fibrillation; CI = confidence interval; CKD = chronic kidney disease; HR = hazard ratio; eGFR = estimated glomerular filtration rate; LVEF = left ventricular ejection fraction.

**Table 4:** Predictors of ERAF.

**Univariate Analysis**		
	**HR (95% CI)**	***p*-value**
Mild to moderate CKD (eGFR 30–59 mL/min/1.73 m^2^) vs. eGFR 60 mL/min/1.73 m^2^ or higher	1.54 (0.94–2.50)	0.084
Female gender	0.94 (0.66–1.35)	0.755
Age at first ablation (years) ≥ 65	0.95 (0.68–1.32)	0.746
Paroxysmal AF	0.50 (0.36–0.69)	<0.001
Months from first episode of atrial arrhythmia > 12 months	1.37 (0.92–2.02)	0.120
Number of tested AAD ≥ 2	1.59 (1.14–2.22)	0.007
Underlying heart disease	1.28 (0.89–1.86)	0.185
Hypertension	0.79 (0.58–1.10)	0.163
CHA_2_DS_2_VASc ≥ 1 (male) or ≥2 (female)	0.92 (0.64–1.32)	0.660
LVEF (%, continuous)	0.98 (0.96–1.00)	0.112
**Multivariable analysis**		
Mild to moderate CKD (eGFR 30–59) vs. eGFR 60 mL/min/1.73 m^2^ or higher	1.56 (0.92–2.63)	0.099
Female gender	0.90 (0.61–1.32)	0.581
Age at first ablation (years) ≥ 65	0.86 (0.59–1.23)	0.401
Paroxysmal AF	0.48 (0.34–0.68)	<0.001
Months from first episode of atrial arrhythmia > 12 months	1.56 (1.11–2.19)	0.011

Legend: AAD = antiarrhythmics drug; AF = atrial fibrillation; CI = confidence interval; CKD = chronic kidney disease; HR = hazard ratio; eGFR = estimated glomerular filtration rate; LVEF = left ventricular ejection fraction.

## Data Availability

The data that support the findings of this study are available from the corresponding author upon reasonable request.
